# A Residual Network and FPGA Based Real-Time Depth Map Enhancement System

**DOI:** 10.3390/e23050546

**Published:** 2021-04-28

**Authors:** Zhenni Li, Haoyi Sun, Yuliang Gao, Jiao Wang

**Affiliations:** 1 College of Information Science and Engineering, Northeastern University, Shenyang 110819, China; lizhenni@ise.neu.edu.cn (Z.L.); haoyisun@outlook.com (H.S.); 2 College of Artificial Intelligence, Nankai University, Tianjin 300071, China; gaoyuliang@mail.nankai.edu.cn

**Keywords:** depth map enhancement, residual network, FPGA, ToF

## Abstract

Depth maps obtained through sensors are often unsatisfactory because of their low-resolution and noise interference. In this paper, we propose a real-time depth map enhancement system based on a residual network which uses dual channels to process depth maps and intensity maps respectively and cancels the preprocessing process, and the algorithm proposed can achieve real-time processing speed at more than 30 fps. Furthermore, the FPGA design and implementation for depth sensing is also introduced. In this FPGA design, intensity image and depth image are captured by the dual-camera synchronous acquisition system as the input of neural network. Experiments on various depth map restoration shows our algorithms has better performance than existing LRMC, DE-CNN and DDTF algorithms on standard datasets and has a better depth map super-resolution, and our FPGA completed the test of the system to ensure that the data throughput of the USB 3.0 interface of the acquisition system is stable at 226 Mbps, and support dual-camera to work at full speed, that is, 54 fps@ (1280 × 960 + 328 × 248 × 3).

## 1. Introduction

Recently, with the proposal of depth map acquisition methods such as structured light method [1] and time-of-flight (ToF) method [2], various consumer-image sensors have been developed, such as the Microsoft Kinect and time-of-flight cameras. Meanwhile, depth maps have also been extensively studied. Depth map is the most effective way to express the depth information of 3D scenes, and is used to solve problems such as target tracking [3], image segmentation [4], and object detection [5]. It is widely used in the emerging applications, such as virtual reality, driving assistance and three- dimensional reconstruction. However, the depth image obtained through sensors is often unsatisfactory, and this will adversely affect back-end applications. Therefore, low resolution and noise interference have become important issues in depth map research.

Many researchers have proposed various methods to reconstruct high-quality depth maps. The depth map enhancement algorithm focuses on repair and super-resolution (SR). General depth map repair algorithms have adopted the method of joint filtering. The methods of depth map SR can be divided into three categories: (1) multiple depth map fusion; (2) image-guided depth map SR; (3) single depth map SR [6]. Xu et al. [7] fused depth maps through multi-resolution contourlet transform fusion, which not only retains image contour information but also improves the quality of the depth map. Li et al. [8] introduced synchronized RGB images to align with the depth image, and preprocessed the color image and the depth image to extract the effective supporting edge area, which ensured the edge details of the image while repairing the depth map. Zuo et al. [9] proposed a frequency-dependent depth map enhancement algorithm via iterative depth guided affine transformation and intensity-guided refinement, and improved performances based on qualitative and quantitative evaluations are demonstrated.

To approximate the real depth map, it is hard to train the depth map to be repaired directly, however, if the jump structure of residual network (ResNet) is adopted, then the training goal becomes to approximate the difference, which is between the depth map to be repaired and the real depth map. Besides, the most widely used implementation platforms are graphics processing unit (GPU) and the field-programmable gate array (FPGA). GPUs support parallel computing, which is essential to achieve real-time performance, although they are very power-consuming and therefore not suitable for embedded applications. In contrast, FPGA is able to support stereo vision system computations with lower power consumption and cost. Therefore, FPGA design for stereo vision systems has become an active research topic in recent years.

In this paper, we propose a depth map enhancement algorithm based on residual network and introduce the FPGA design and implementation for depth sensing. The network uses a fully convolutional network, which eliminates the preprocessing process in the existing algorithm and deepens the network structure. For the problems in deep network training, a batch standardization and residual structure is proposed. Our method uses peak signal to noise ratio (PSNR) and root mean square error (RMSE) for evaluation. Experiments show that the system has reached the predetermined performance index. In summary, the main contributions of this paper are as follows:

(1) We design a dual-camera synchronous acquisition system based on FPGA and collect intensity map and depth map at the same time as the input of neural network.
(2) We propose a depth map enhancement algorithm based on residual network, extracting features from the acquired intensity map and depth map in two ways, and then performing fusion and residual calculation. The result shows our proposed network has better performance than existing low-rank matrix completion (LRMC) [10], denoise and enhance convolutional neural network (DE-CNN) [11] and data-driven tight frame (DDTF) [12] algorithms on standard dataset.
(3) We have completed the test of the system to ensure that the data throughput of the USB interface of the acquisition system is stable at 226 Mbps and support dual-camera to work at full speed, 54 fps@ (1280 × 960 + 328 × 248 × 3).

However, some limitations of our proposal are as follows:

(1) The acquisition system consists of multiple development boards, which are bulky and not flexible enough, so it is necessary to design an all-in-one board, which concentrates the FPGA, TC358748, CYUSB3014 and other chips in the acquisition system on one printed circuit board (PCB).
(2) The acquisition program runs on the central processing unit (CPU), but the current single-threaded sequential processing will consume a lot of time, and the system will become less real-time.

The rest of this paper is organized as follows. In Section 2, we discuss the related work including the residual network design and FPGA design of image acquisition system. In Section 3, the details of the proposed algorithm are presented. Details of hardware architectures and system implementation are shown in Section 4. In Section 5, the experimental results are given in terms of the enhanced performance indicators of the depth map. Finally, we conclude this paper in Section 6.

## 2. Related Work

### 2.1. Residual Network Design

High quality depth images are often applied in the field of computer vision, and many works introduce deep learning into various image processing applications, such as depth map enhancement. Ni et al. [13] proposed a color-guided convolutional neural network method for depth map super resolution. They adopted a dual-stream convolutional neural network, which integrates the color and depth information simultaneously, and the optimized edge map generated by the high-resolution color image and low-resolution depth map is used as additional information to refine the object boundary in the depth map. The algorithm can effectively obtain depth maps, but cannot take the low super resolution depth map and high super resolution color image as inputs and directly outputs the high super resolution depth map. Zhou et al. [14] proposed a deep neural network structure that implements end-to-end mapping between low-resolution depth maps and high-resolution depth maps and proved that deep neural networks were superior to many state-of-the-art algorithms. Chen et al. [15] proposed a single depth map super-resolution method based on convolutional neural networks. Super-resolution is achieved by obtaining high-quality edge maps from low-quality depth images, thereby using high-quality edge maps as the weight of the regularization term in the total variation (TV) model. Korinevkava and Makarov [16] proposed two deep convolutional neural networks to solve the problem of single depth map super-resolution. This method has good performance on the RMSE and PSNR indicators and can process depth map super-resolution in real time with over 25–30 frames per second rate. However, their method cannot be applied to other datasets because the quality of the results varies heavily.

Li et al. [17] proposed an end-to-end convolution neural network (CNN) combined with a residual network (ResNet-50) to learn the relationship between the pixel intensity of RGB images and the corresponding depth map. He et al. [18] developed a model of a fully connected convolutional auto-encoder where the middle layer information was also used to estimate the depth. Kumari et al. [19] has developed a convolutional neural network encoder decoder architecture and their model integrates residual connections within pooling and up-sampling layers, and hourglass networks which operate on the encoded feature. Siddiqui et al. [20] proposed a deep regression network using a transfer learning approach, where an encoder is used to initialize it to extract dense features and a decoder is used to upsample and predict the desired depth. Schlemper et al. [21] proposed a framework for reconstructing sequences images from undersampled data using a deep cascade of convolutional neural networks to accelerate the data acquisition process. Li et al. [22] treated the depth estimation problem as a construction from a sequence-to-sequence model, using location information and attention, with dense pixel matching instead of cost volume.

Compared with existing works, CNN is a basic architecture in depth map enhancement, and some algorithms added encoder and decoder, adopted some ResNet modules in the hidden layers of the network, or used sequence-to-sequence model. This paper designs a residual network, which uses dual channels to process depth maps and intensity maps respectively, then calculates the residual of depth maps and the output of network and cancels the preprocessing process. For the function of depth map restoration, this algorithm improves PSNR by 3 dB. For the super-resolution function of depth map, this algorithm reduces RMSE by more than 10 times. In addition, the processing speed of this algorithm is also greatly improved, because it does not need CPU to participate in the calculation, so GPU can be used to achieve good acceleration effects.

### 2.2. FPGA Design of Image Acquisition System

With the increasing non-repetitive engineering (NRE) cost and design cycle of application specific integrated circuit (ASIC), FPGA design of stereo vision has gradually become an active research topic in recent years [23]. FPGAs are able to find an optimal trade-off between performance, energy efficiency, fast development and cost, therefore, they are widely adopted in neural network applications [23,24,25]. And FPGA design of stereo vision systems has become an active research topic in recent years.

With the progress of the stereo correspondence algorithms [26,27], a large number of FPGA architectures have been proposed. Dong et al. [28] designed the image acquisition and processing system of the color sorter based on FPGA. They completed the construction of the hardware platform and the design of the image processing algorithms. Their experiment confirmed that the system based on FPGA had high-speed and high-precision performance. Manabe et al. [29] proposed a real-time processing system for super-resolution moving images based on CNN. The system can perform super-resolution between 960 × 540 and 1920 × 1080 at 60 fps. Shandilya et al. [30] applied image enhancement based on FPGA to the automatic vehicles number plate (AVNP) problem and achieved image enhancement by estimating the best value of various aspects of image quality. Prashant et al. [31] processed blurred or useless image information on a FPGA SPARTAN 3 XC3S500E board and obtained high-quality images by image fusion. However, the detailed FPGA implementation is not described. In contrast, Pfeifer et al. [32] implemented an active stereo vision system on a Xilinx Zynq-7030 SoC. The system provides a computation speed of 12.2 fps, at a resolution of 1.3 megapixel, but the system cannot deal with higher resolutions. Lee et al. [33] designed a stereo matching accelerator that achieved 41 fps@480 × 270 performance on the KU040 FPGA board, but it could not deal with high-resolution images and needed to be converted to 480 × 270 resolution.

Compared with the above mentioned system, a synchronous real-time acquisition system of dual cameras based on FPGA is implemented, which can accurately control ToF camera and visible light camera to acquire images synchronously, and transmit the data to the back-end acceleration platform in real time through USB3.0 interface. The FPGA design in this paper has a certain improvement in computing speed and depth map quality. In addition, the data throughput of the USB interface in the system proposed can be stabilized at 226 Mbps, and supports dual cameras to work at full speed.

## 3. Materials and Methods

In this section, we have proposed a CNN-based model to estimate the depth information from the single image by integrating the residual depth image.

### 3.1. CNN Architecture and ResNet

CNN architectures usually include a contractive part that progressively reduces the resolution of the input image through a series of convolution and pooling operations, however, in regression problems, if the output is expected to be a high-resolution image, some form of upsampling is required to obtain a larger output map. However, with the increasing number of network layers, a degradation problem might occur. ResNet [34] is a deep convolutional network proposed in 2015, which addressed the degradation problem.

**Figure 1:**
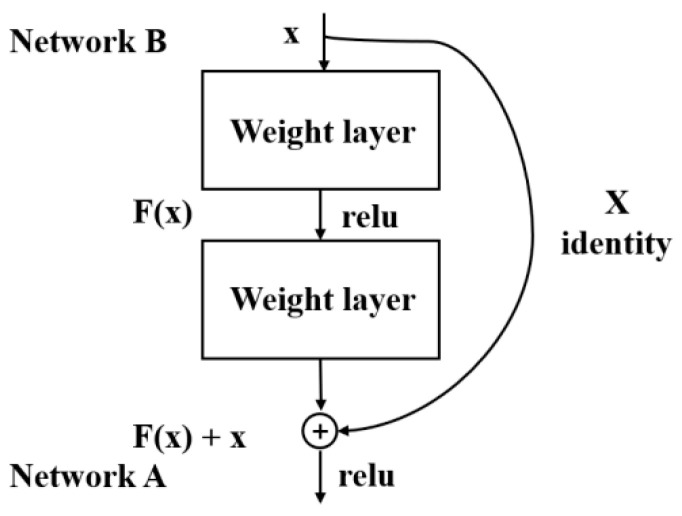
Shortcut connection.

As shown in Figure 1, ResNet uses shortcut connection as the basic structure of the network, and the output of network A can be defined as H(x)=F(x)+x, where x denotes the output of network B and F(x) represents the residual mapping to be learned. After adding the identity mapping, if F(x) converges to 0, then, which means that network A achieves almost the same effect as network B.

In Inception-v4 [35], the author combined the residual and inception structures and found that residual was able to accelerate the training of the inception network with a small improvement in accuracy. And in this paper, we design a CNN network combined with a residual network, and specific improvements will be discussed in the next section.

### 3.2. Proposed Neural Network Design

**Figure 2:**
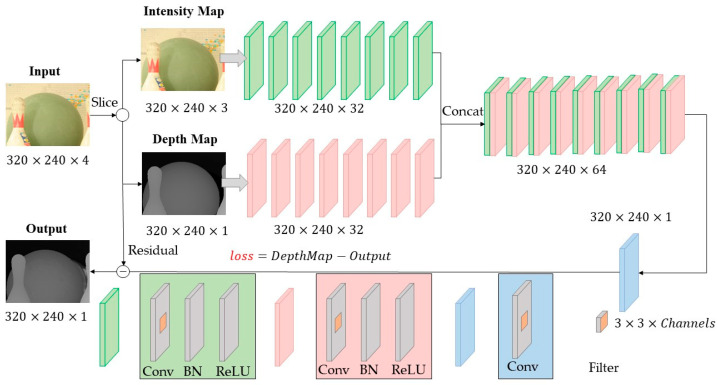
The proposed network structure.

The network structure designed in this paper is shown in Figure 2. The input is the intensity map and depth map, referring to the full convolutional and multi-branch design idea of GoogleNet [35]. The intensity map and depth map are input to the two convolutional networks separately to extract the feature maps, followed by fusion and residual calculation. Compared with DE-CNN [11], there are four major improvements in our network structure:

1. Dividing the depth map and intensity map into two separate channels, which is different from DE-CNN where the depth map and intensity map are placed in the two channels of the input image;
2. Removing the pooling layer, which enables more accurate feature maps to be extracted;
3. Deepening the network structure, which provides better representation than the shallow network in DE-CNN. Meanwhile, to address the problem of gradient vanishing and degradation problem when training deep neural networks, the batch normalization and residual structure are adopted;
4. Remove pre-processing. CNN has a more robust performance compared to conventional algorithms.

In this paper, we adopt the Caffe framework to implement the network as shown in Figure 2. The basic data structure of Caffe is a four-dimensional array called Blob, in order from outside to inside, starting from N (the number of input images per batch, axis = 0), C (the number of channels of the image, axis = 1), H (the height of the image, axis = 2) and W (the width of the image, axis = 3), i.e., N×C×H×W. We place the depth data in channel 0 and the intensity data corresponding to the depth data in channel 1. Thus, the data is first split from the C dimension (axis = 1) using the Slice layer provided by Caffe. And after extracting the features of the depth map and the intensity map separately, the two parts of the feature map are connected along the C dimension (axis = 1) using the Concat layer.

As shown in Figure 2, the convolutional layers can be divided into 2 categories. The first class of convolutional layers consists of 4 parts: convolution, bias, batch normalization and ReLU activation function. Assume that in the lth convolutional layer, the ith output feature map and its corresponding bias terms are $q_{i}^{l}$ and $b_{i}^{l}$, the jth input feature map is $p_{j}^{l}$, and the feature map pair $\left(q_{i}^{l},p_{j}^{l}\right)$ corresponds to the convolution kernel $W_{ij}^{l}$.The first class of convolutional layers can be represented as:

(1) $$q_{i}^{l}=f\left(BN_{\gamma ,\beta }\left(\sum_{j}\left(W_{ij}^{l}*p_{j}^{l}\right)+b_{i}^{l}\right)\right)$$

The second class of convolutional layer consists of convolution and bias operations only, without batch normalization and ReLU activation functions. The second class of convolutional layers can be represented as:

(2) $$q_{i}^{l}=\underset{j}{\sum }\left(W_{ij}^{l}*p_{j}^{l}\right)+b_{i}^{l}$$

It is very difficult to directly train the depth map to be repaired to approximate the real depth map. However, if the structure of ResNet is adopted, the training goal becomes to approximate the difference between the depth map to be repaired and the real depth map, which needs to be normalized to [0,1] before the image is input to the network, so the approximate difference is relatively small, and empirically it is easier for ResNet to train such data successfully.

According to Inception-v4 Network, some of the convolution kernels are 1×1, 3×3, and in paper [36], the author argues two 3×3 convolution filters are equivalent to one 5×5 convolution filter, with fewer parameters than one 5×5 convolution filter and make network deeper and extract more complex features. So 3×3 convolution filter is adopted in this architecture. Besides, the image is padded with zero to make the output image the same size as the input image. And there are three similarities between the settings of the above two classes of convolutional layers:

(1) The size of the convolution kernel is 3 × 3;
(2) The strides are set to one and the edges are padded with zero;
(3) MSRA [31] initialization is applied.

Assuming that the input is a feature vector $X=\left(x_{1},x_{2},\ldots x_{m}\right)$, and the output feature vector is $Y=\left(y_{1},y_{2},\ldots y_{m}\right)$, with the scaling factor γ and the translation parameter β initialized to 1 and 0 respectively, then the batch normalization can be described as Algorithm 1.

**Algorithm 1**. Compute Batch Normalization. Default settings are γ=1,  β=1, λ=0.99, ϵ=0.01**Input:** The feature vector $X=\left(x_{1},x_{2},\ldots x_{m}\right)$ **Output:** The feature vector $Y=\left(y_{1},y_{2},\ldots y_{m}\right)$ 1: **function** Batch normalization (X) 2:  $\mu_{X}\leftarrow \frac{1}{m}\overset{m}{\underset{i=1}{\sum }}x_{i}$ (Calculate the mean of Batch) 3:  $\sigma_{X}^{2}\leftarrow \frac{1}{m}\overset{m}{\underset{i=1}{\sum }}{\left(x_{i}-\mu_{\beta }\right)}^{2}$ (Calculate the variance of Batch) 4:  $S_{t+1}\leftarrow \lambda S_{t-1}+\left(1-\lambda \right)S_{t}$ (Update the sliding mean and variance) 5:  $X^{\prime }\leftarrow \frac{X-\mu_{X}}{\sqrt{\sigma_{X}^{2}+\varepsilon }}$ (Normalize Z-score) 6:  $Y\leftarrow \gamma X^{\prime }+\beta$ 7:  **return** Y8: **end function**

The batch normalization in Caffe consists of two parts, the BatchNorm layer and the Scale layer, so the first class of convolutional layers requires five Caffe-defined layers for implementation including Conv, Bias, BatchNorm, Scale and ReLU, while the second class of convolutional layers requires only two Caffe-defined layers, Conv and Bias.

The BatchNorm layer corresponds to line 2–5, and the Scale layer corresponds to line 6. During the training process, the BatchNorm layer runs in line 2–5, and in addition to calculating the sliding mean and sliding variance, the scaling factor γ and the translation parameter β are updated according to the gradient descent algorithm, and these four parameters are stored in the Caffemodel file after training. Instead, line 2–4 are ignored in the test BatchNorm layer and step 5 is calculated directly using the sliding mean and sliding variance stored in the Caffemodel file, which avoids depending on the Batch Size during testing and improves the generalization ability of the network.

### 3.3. Dataset and Network Training

Binary large object (BLOB) is the standard array structure and unified storage interface for the entire framework. Caffe uses Blob to store, exchange and manipulate information, its data type is a four-dimensional array. Encoded image formats such as PNG cannot be used directly. Instead, Caffe supports three data structures including lightning memory-mapped database (LMDB), level data base (LevelDB) and hierarchical data format (HDF-5), which can convert training samples and labels, according to image size, Batch Size and number of samples, into a four-dimensional array corresponding to the Blob format and integrate them into a single file. In this paper, HDF-5 is adopted.

**Figure 3:**
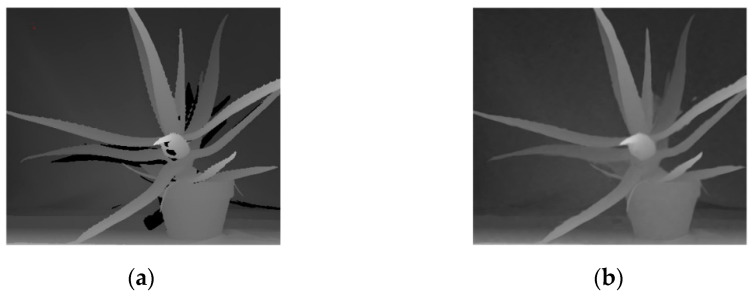
The Aloe scene in Middlebury data set: (**a**) original; (**b**) processed by LRMC.

Two depth datasets, Middlebury and MPI Sintel [37], are used to provide the training and test sets. The Middlebury dataset is a depth map taken from the real world using structured light, which allows for better training of the network, and is also used as a benchmark when comparing the performance of various depth map restoration algorithms. However, as shown in Figure 3a, the depth map in the dataset has some holes, which will affect the training of the restoration function, so we need to pre-process the images in this dataset.

LRMC was a better depth map restoration algorithm, and Figure 3b shows the results of the LRMC algorithm for the Aloe scene in the Middlebury dataset. Although Si Lu’s team did not make the code publicly available, the Middlebury dataset processed by the LRMC algorithm, including 30 sets of RGBD images, is published on the website. In this paper, 24 of these images were selected for the training set and 2 for the test set. However, the training sets produced with these images could only train the network up to the level of the LRMC algorithm at best. Comparing Figure 3a,b, LRMC algorithm blurs the image edge details, causing some deviation in the correct depth values. The depth map in the MPI Sintel data set is obtained from the 3D micro animation Sintel using the optical flow method. Although it is not like the Middlebury data set which can restore the real scene, it has the advantage of being accurate and free of holes. In order to compensate for the degraded accuracy of the LRMC-processed depth maps, 64 sets of RGBD images from the Sintel dataset were selected to supplement the training set, and another 4 sets were selected to supplement the test set. From a data-driven perspective, such a training set allows the network to learn the features of real scenes while considering accuracy issues.

To be specific, Ground Truth consists of two parts: 26 depth maps from the Middlebury dataset processed by LRMC algorithm and 68 original depth maps from the Sintel dataset, which need to be normalized. And the depth map used in the sample was not processed by LRMC algorithm. The following methods are adopted to add noise and holes to the original Y-D image: the selected RGB image is first converted to a greyscale map and normalized into the range of [0,1], and then Additive White Gaussian Noise (AWGN) is added as the Y channel of the input sample; next the original depth map is normalized into the range of [0,1], and 13% of the pixels of this image are randomly set to 0 as the D channel of the input sample.

**Figure 4:**
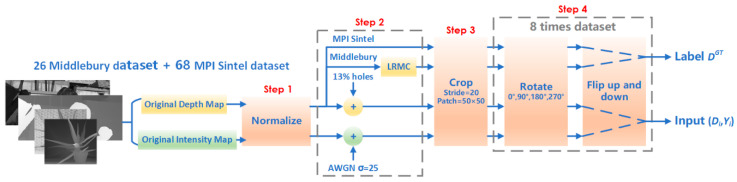
Data augmentation.

Deep learning requires training samples of at least $10^{4}$ orders of magnitude, therefore a simple but efficient data augmentation method is applied to guarantee sufficient training samples. To be specific, as shown in Figure 4 for Ground Truth and noise-added Y-D images, the data augmentation in the training process is implemented through flipping left to right operation, along with rotation 90° in up, down, left and right four directions, so we can get 8 times the data set. Then the stride is set to 20 with 50×50 patches and discard patches with lack strides. Finally, there are 400,512 sets of Ground Truth and training samples in the training set, and 2590 sets of Ground Truth and test samples in the test set. During the training, we input a batch sample with a batch size of 128 per iteration, and iterate all batches per epoch.

The Adam gradient descent algorithm in Caffe Solver is adopted in this paper, and the parameters $\beta_{1}$, $\beta_{2}$ and ϵ use the default values in the literature [38], i.e., $\beta_{1}=0.9$, $\beta_{2}=0.999$ and $\epsilon =10^{-8}$. The base learning rate is fixed at $10^{-5}$. Different from Caffe model which sets the learning rate of the bias term to twice the learning rate of the weights, the additional learning rate of the weights is set to 1 and the learning rate of the bias term is set to 0.1 in this paper.

Assuming that M denotes the number of samples, $D_{i}^{GT}$ denotes ground truth, W denotes the weight, and $f\left(D_{i},Y_{i},W\right)$ denotes the forward function of the whole network, we can mathematically describe the MSE loss term as follows:

(3) $$L\left(W\right)=\frac{1}{M}\overset{M-1}{\underset{i=0}{\sum }}\frac{1}{2}∥f\left(D_{i},Y_{i},W\right)-{(D_{i}-D_{i}^{GT})∥}^{2}$$

By adding the L2 regular term, where the default decay weight is set to $5\times 10^{-4}$, the MSE error can be denoted as:

(4) $$J\left(W\right)=L\left(W\right)+\lambda {∥W∥}^{2}$$

The server CPU model is an Intel Core i7 6700K with 64 GB of random-access memory (RAM) and the GPU model is NAVIDA GTX1080 with 8 GB of video memory. The software environment includes Ubuntu 16.04.4 LTS, Caffe 1.0, CUDA 8.0, CuDNN 6.0.21, TensorRT 4.0.0.3, Python 3.5 and MATLAB 2018a. In addition, two GPUs are required when setting batch size to 128 for Linux terminal training, otherwise an insufficient video memory error is prompted.

**Figure 5:**
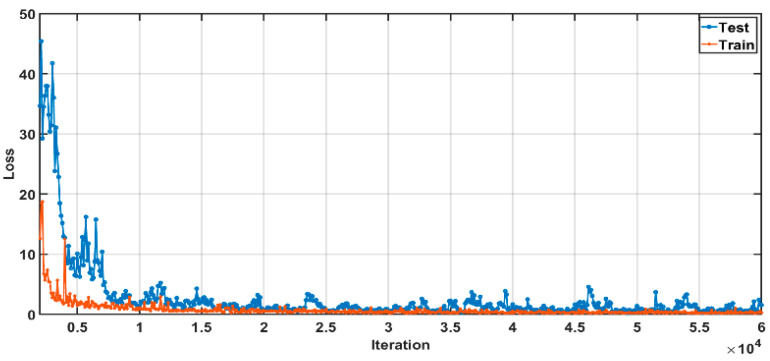
Iteration-Loss curve.

The training error and test error were recorded every 100 iterations and a Snapshot file was saved. After running 60,000 iterations, the Iteration-Loss curve as shown in Figure 5 can be obtained. It can be seen that as the training proceeds, the loss value decreases smoothly and eventually the training and testing errors stabilize around 0.5. The best performance was tested for the 56,000th iteration, and the Caffemodel file from this iteration will be used to test the performance of the network in the next section.

## 4. Full-Pipeline FPGA Implementation

### 4.1. Hardware Architecture Overview

**Figure 6:**
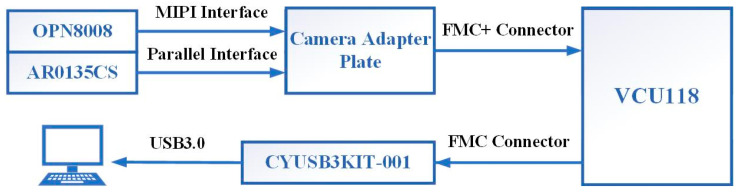
Physical connection diagram of the acquisition system.

The physical connection is shown in Figure 6. The USB 3.0 controller CYUSB3014 [39], is a chip that internally integrates the physical layers of USB 2.0 and USB 3.0, the 32-bit microprocessor ARM926EJ-S, and general programmable interface II (GPIF II), a second-generation general-purpose programmable interface for communication with microcontrollers, FPGAs or image sensors. An ON Semiconductor AR0135CS was selected as the sensor for the visible camera and OPN8008 of OPNOUS was selected as the sensor for the ToF camera. The camera serial interface 2 (CSI-2) interface used by the OPN8008 is a high-speed differential serial interface for camera data transmission under the mobile industry processor interface (MIPI) interface.

**Figure 7:**
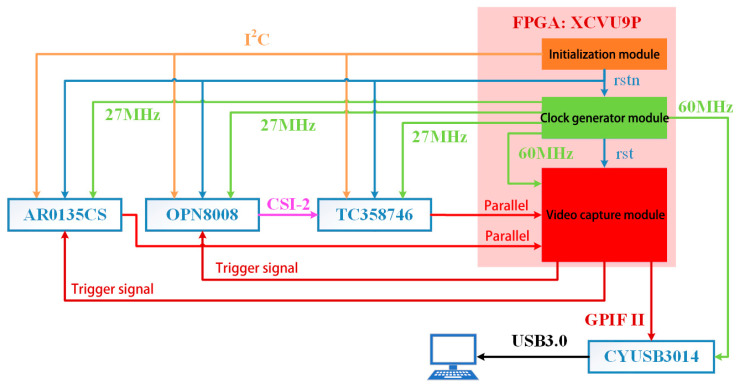
Top-level design of FPGA.

Then an overview of the proposed FPGA system is discussed here. As shown in Figure 7, the design of the FPGA mainly includes the initialization module, the clock generation module and the video capture module. The clock generation module is implemented in FPGA, which uses the VCU118 on-board crystal as the clock source and the FPGA on-chip clock management module to generate the clock, providing a 27 MHz reference clock for the AR0135CS, TC358748 and OPN8008, and a 60 MHz DMA clock for the GPIF II interface.

### 4.2. Initialization Module

**Figure 8:**
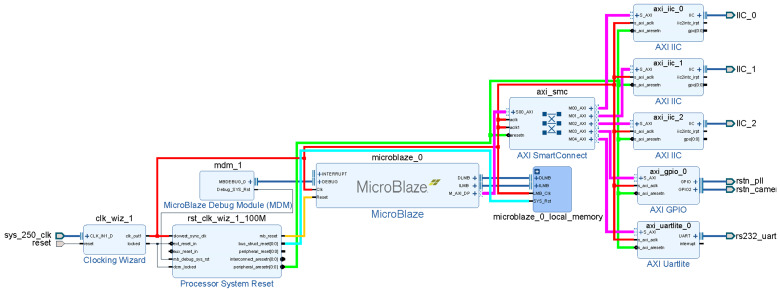
Initialization module.

**Figure 9:**
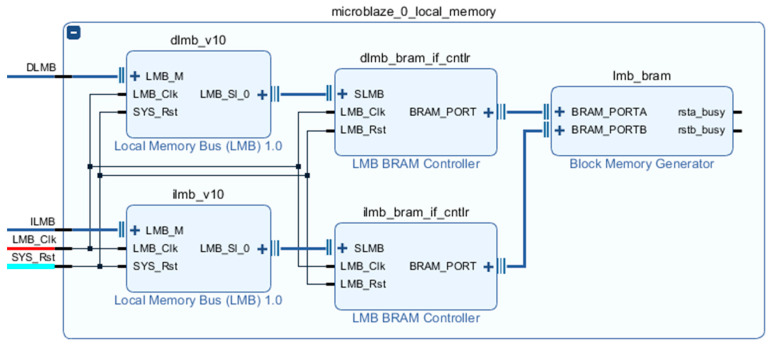
Internal structure of local memory.

The core of the initialization module is MicroBlaze Soft Processor Core, and the system connection is shown in Figure 8. A local memory with a capacity of 64 KB is used as the processor running memory, and its internal structure is shown in Figure 9.

The AXI4 bus and all intellectual property (IP) cores are 100 MHz clock drivers generated by the clock wizard clk_wiz_1. clk_wiz_1 is set to high level and the reset pin is locked to the CPU_RESET button on the VCU118; the clock source is the 250 MHz differential clock source on the board VCU118.

A MicroBlaze core has only one set of AXI4 master ports, as shown in Figure 9. M_AXI_DP is the data channel, while the command channel M_AXI_IP is not used at this time. Xilinx provides AXI SmartConnect IP cores for mounting multiple devices on the AXI4 bus. In this design, three AXI IIC IP cores are mounted on the AXI4 bus for inter-integrated circuit (I^2^C) communication, one AXI general-purpose input/output (GPIO) IP core is used to generate reset signals for external modules and chips, and one AXI Uartlite IP core is used to print the operating status from the serial port.

The XGpio_Initialize function is used to initialize the GPIO core, then XGpio_DiscreteWrite is used to pull up rstn_pll to unlock the reset of the clock generation module. Since the clock has been stabilized after a delay of 50 μs, the rstn_camera is pulled up again at this time to unlock the reset of the image sensor and TC358748, finally completing the power-up process of the external chip.

The initial configuration of the three external chips, including OPN8008, is completed by operating the corresponding AXI IIC IP cores. In this paper, the I^2^C core operates in the default 100 KHz standard mode, and we complete the I^2^C writing operations by writing byte sequences to three registers 0x100, 0x108 and 0x120 through the Xil_Out32 function [40]. The register values and addresses of OPN8008 are 8 bits, and that of AR0135CS and TC358748 are 16 bits.

All three chips have software to generate the configuration and the byte sequence according to the selected function, and then operate the I^2^C core as described above to send the generated bytes out. The configuration of the Register Wizard software provided by ON Semiconductor shows that the image size and frame rate of the AR0135CS is 1280×960@54fps. Since the AR0135CS is required to work in trigger mode in this paper, it also has to write 0x19D8 to the 0x301A register, which is not included in the byte sequence generated by the software.

### 4.3. Clock Generation Module

**Figure 10:**
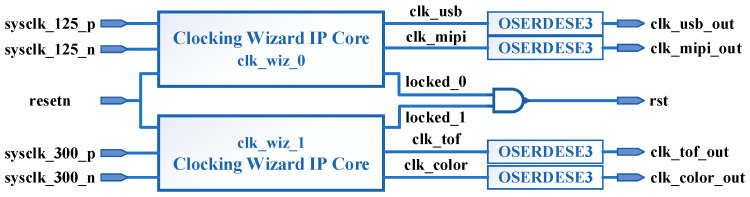
Clock generator schematic.

The schematic of the clock generation module is shown in Figure 10. The clock source of clk_wiz_0 and clk_wiz_1 both use the on-board crystal of VCU118. The reset input *resetn* is active low and is driven by the initialization module. The output of the IP core *locked* is active high and *locked* is set to 1 when the phase-locked loop output is stable. The outputs of the Not And (Nand) operation of locked_0 and locked_1 are used as the high active reset signal *rst* for the video capture module. *clk_usb* is the 60 MHz Direct Memory Access (DMA) clock used to drive the video capture module and GPIF II interface. *clk_tof*, *clk_mipi* and *clk_color* are the 27 MHz reference clocks for OPN8008, TC358748 and AR0135CS.

The output double data rate register (ODDR) is located in the IOB of FPGA, and sending the above 4 clocks through ODDR is good for synchronization of clocks and data on the bus. However, for the three external chip reference clocks such as *clk_tof*, the use of ODDR or not has almost no effect on the performance. The OSERDESE3 (Output SerDes, Output Serializer) in Figure 10 is a specific implementation of ODDR in the UltraScale+ family of chips [41]. The simulation result of the clock generation module shows that the outputs of both PLLs are stable after about 6.692 μs.

### 4.4. Video Capture Module

**Figure 11:**
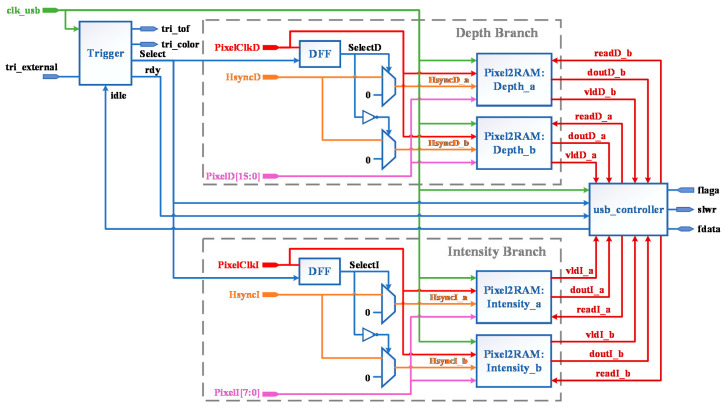
Video capture module.

The structure diagram of the video capture module is shown in Figure 11, including three sub-modules, that is, Trigger, Pixel2RAM and usb_controller. Among them, the Pixel2RAM module is the storage controller, and there are two instances of Depth_a and Depth_b in Depth Branch, which is responsible for storing the depth data from the parallel port input of TC358748 into UltraRAM. PixelClkD, HsyncD and PixelD are pixel clock, field synchronization and pixel data respectively.

In this paper, ping-pong method is proposed to improve the operation speed, and the chip select signal *Select* is generated by the Trigger module. Since the Trigger module is driven by *clk_usb clock*, the SelectD output from the DFF (D Flip-Flop) is in the *PixelClkD* clock domain after a beat of *Select* with *PixelClkD*. *SelectD* and its inverse signal control a data selector, and when *SelectD* is high, *HsyncD_a* is driven by *HsyncD* and *HsyncD_b* is pulled low, so the pixels of the current frame will be written to Depth_a, while the usb_controller module reads the pixels of the previous frame from Depth_b and sends them to the CYUSB3014 chip through the GPIF II interface; conversely, when *SelectD* is low, Depth_a reads and Depth_b writes.

The intensity branch is responsible for receiving data from the parallel port of AR0135CS, and its design is similar to the depth branch. The design of the three seed modules Pixel2RAM, Trigger and usb_controller will be introduced in the following sections.

#### 4.4.1. Pixel2RAM Sub-Module

**Figure 12:**
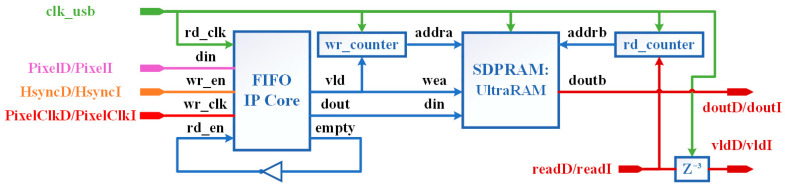
Pixel2RAM sub-module.

Figure 12 shows the architecture of Pixel2RAM submodule. An asynchronous first in first out (FIFO) is first used to transfer PixelD/PixelI from PixelClkD/PixelClkI clock domain to clk_usb clock domain. The FIFO is implemented using Xilinx FIFO Generator IP core and the configuration information is listed in Table 1.

The 8-bit FIFOs are used in Intensity_a and Intensity_b in Figure 11, and the 16-bit FIFOs are used in Depth_a and Depth_b. And the bit width of all FIFOs’ read port is 32 bits, which is designed to accommodate the GPIF II interface bit width. We invert the read null signal empty from the IP core output and use it as input to the read enable signal *rd_en*, so that the data will be read automatically when the FIFO is non-empty. The word order is given in [42] when the write-read bit-width ratio is 1:4 for 2-bit data input, while for 32-bit data read, the input is replaced with 8 bits, then the 32-bit data read is in big endian order.

The UltraRAM implementation of simple dual port RAM (SDPRAM) can only be called by a Xilinx Parameterized Macro (XPM). The data of SDPRAM can only be written from port A and be read from port B, so there are only write enable signal *wea* and no read enable signal. There are two limitations when SDPRAM is implemented with UltraRAM: (1) Ports A and B have to use common clock, so switching the clock domain with asynchronous FIFO is necessary in this design; (2) the read delay is at least 3 clock cycles, so we have to shift the input read signal from usb_controller *readD/readI* by 3 clk_usb cycles and use it as the valid signal *vldD/vldI* for doutb, in fact Z^−3^ in Figure 12 is the shift register with a depth of 3.

#### 4.4.2. Trigger Sub-Module

**Figure 13:**
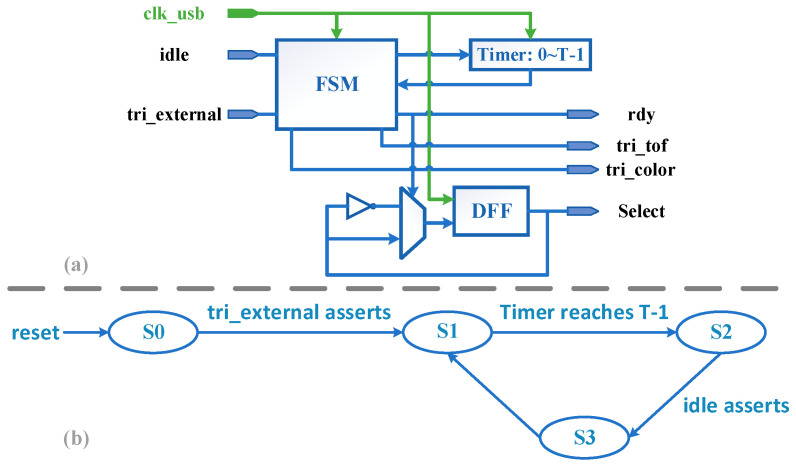
Trigger sub-module. (**a**) Structure, (**b**) FSM state transfer diagram

The Trigger sub-module is responsible for the control of the ping-pong operation and the generation of the image sensor trigger signal, and the core is a Finite State Machine (FSM). The structure of the module and the FSM state transfer diagram are shown in Figure 13a,b.

S0 is the standby state, and when the external trigger signal *tri_externl* is detected high, the FSM is transferred from S0 to S1, and the capture module is activated and starts to run. Besides, note that the state values are encoded by the one-hot code, S0–S3 are 0001/0010/0100/1000 respectively. In this design, *tri_externl* is connected to the button for the first time triggering the camera after power on. S1 is used to achieve the preset frame rate and complete the trigger. The operating frame rate of an image sensor in trigger mode must be greater than the trigger signal frequency to ensure that the sensor can process and send a frame within the trigger signal period. In this paper, the operating frame rate of AR0135CS is 54 fps and the frequency of the trigger signal is 30 Hz. S2 is used to wait the usb_controller idle. Signal *rdy* and signal *idle* are a pair of handshake signals. *Idle* is used to indicate whether the usb_controller module has finished the current DMA transfer; *rdy* is held for only one clock cycle and is used to inform the usb_controller module that the new pixel data is ready. S3 is used to enable the signal *rdy* and to invert the signal *Select*, and this state is held for only one period.

#### 4.4.3. USB_Controller Sub-Module

**Figure 14:**
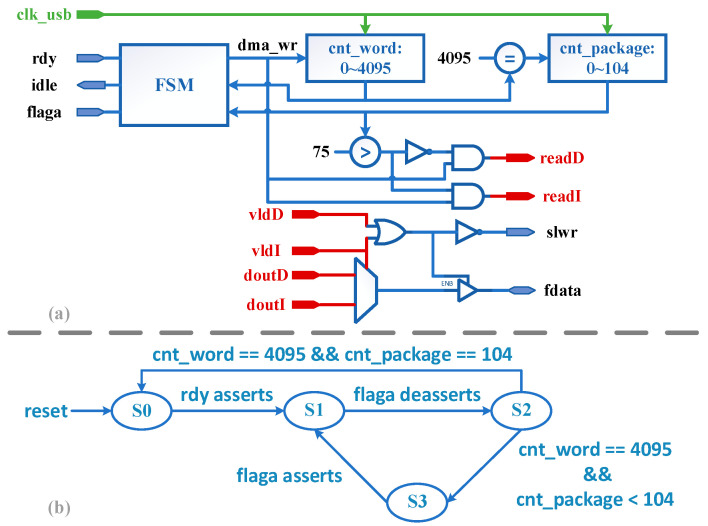
USB_controller sub-module. (**a**) Structure, (**b**) FSM state transfer diagram

The structure of the USB controller module and the finite-state machine (FSM) state transfer diagram are shown in Figure 14a,b. S0 is the idle state, used to complete the handshake with the Trigger module, and signal *idle* is active in this state. When the signal *rdy* is detected high, the FSM shifts from S0 state to S1 state. State S1 is used to wait for the DMA buffer to be empty, and the FSM transitions from S1 to S2 when flaga is pulled high. S2 is the DMA write state. *Dma_wr* is valid only in S1 and is used to drive the SDPRAM read signals *readI* and *readD*. The state transfer in S2 is determined by both the word counter *cnt_word* and the package counter *cnt_package*. When the cnt_word count is full, usb_controller will send 4096 read signals continuously. At this point if the cnt_package count value is not full then the data in RAM is not read, S2 turns to S3 and waits for flaga to be pulled high, then S3 turns back to S1 to continue the cycle. According to the literature [43], flaga does not pull down immediately after the current Buffer *write* is full, but there is a delay of 4 bus clock cycles, so if we enter state S1 directly without waiting, it will definitely lead to a misjudgment of the Buffer empty-full state. If *cnt_package* count is full, all data is read and S2 goes back to S0 to wait for the next round of transfers. Besides, write enable signal *slwr* of DMA is driven by the Nand operation of data valid signals *vldI* and *vldD* given by the Pixel2RAM module, without the involvement of the FSM.

### 4.5. FPGA Resources Analysis

FPGA resources utilization are shown in Table 2. From the results, we could find that with an efficient design of the system, the resource utilization of FPGA is acceptable. For example, the utilization of lookup table (LUT) by the whole system is only 0.36%. Moreover, LUT and block RAMs (BRAM) are also two critical criterions to evaluate the resource utilization. From the results, we could find that BRAM is constrained for the proposed implementation.

## 5. Experimental Results

### 5.1. Experimental Setup

**Figure 15:**
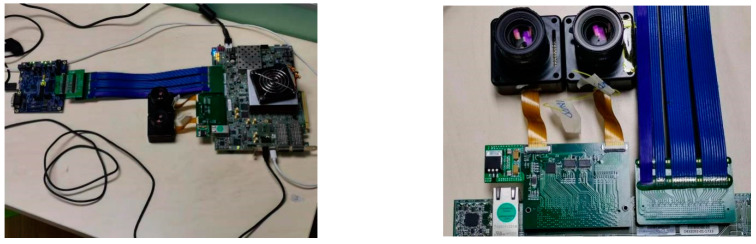
Physical connection diagram of the realized system.

With the FPGA architecture designed above, a real-time system for depth map restoration and super-resolution is implemented. The FPGA used is Xilinx XCVU9P-L2FLGA2104 embedded in VCU118 development. Figure 15 shows the connection of the image acquisition system and the snapshot of cameras and FMC+adaptor. ON Semiconductor’s AR0135CS is selected as the sensor for the visible camera, and OPN8008 from OPNOUS is selected as the sensor for the ToF camera. The main parameters of these two sensors are listed in Table 3.

### 5.2. Result Analysis and Comparison

#### 5.2.1. Depth Map Repair Performance

PSNR is proposed as a measure of depth map restoration performance. It should be noted that the locations with voids are not included in the MSE when calculating the PSNR value. In testing the restoration function, we selected six scenes in the Middlebury dataset and cropped the image size to 320×240, added σ=25 AWGN to the intensity map, and randomly added 5%, 10%, 15%, and 20% voids to the depth map. The PSNR performance before and after restoration is statistically shown in Table 4 and Table 5. It can be seen that the algorithm proposed in this paper can improve the PSNR value by about two times.

LRMC and DE-CNN are the representatives of traditional restoration algorithms and deep learning-based restoration algorithms. A review of literature [10,11,12] shows that the test conditions of these three algorithms are adding σ=25 AWGN to the intensity map and randomly adding 13% voids to the depth map. The comparison of algorithm performance under the same conditions is shown in Table 6, which shows that DE-CNN has no PSNR performance advantage over the traditional algorithm LRMC, while the proposed algorithm in this paper outperforms LRMC, DE-CNN and DDTF in terms of PSNR performance on all six scenarios.

**Figure 16:**
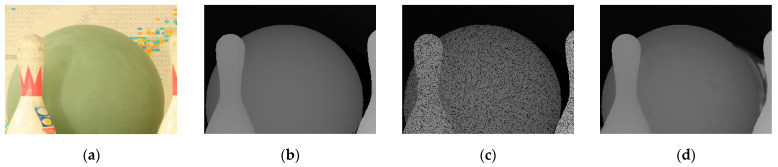
Scene of bowling (**a**) RGB image; (**b**) Original depth image; (**c**) Depth image with 13% holes added; (**d**) Depth image restoration from our method.

**Figure 17:**
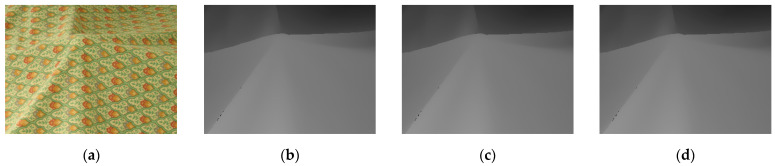
Scene of clothes (**a**) RGB image; (**b**) Original depth image; (**c**) Depth image with 13% holes added; (**d**) Depth image restoration from our method.

Figure 16 and Figure 17 show the results of the depth map enhancement tests for the Bowling 1 and Cloth 1 scenes. These images are: (a) the scene; (b) the original depth map; (c) the depth map with 13% holes added; and (d) the restoration results.

#### 5.2.2. Accuracy

RMSE is proposed as a measure of the super-resolution performance of the depth map, and locations with voids are not counted. In testing the super-resolution function, we selected six scenes in the Middlebury dataset but without cropping, added σ=25 AWGN to the intensity map. The depth map is used without adding noise, and the down-sampling method in literature [44] is used to obtain images with constant field of view but lower resolution, and then the low-resolution depth map is up-sampled to obtain the input of the network.

The RMSE values for up sampling factors of 2 and 4 are given in Table 7 and Table 8, and it can be seen that the performance of the proposed algorithm has been greatly improved compared to the existing algorithms. The main reasons for the improvement are the followings: (1) The input intensity map provides complementary information to make the algorithm more oriented; (2) The representation capability of the convolutional neural network is stronger than that of the conventional algorithm based on convex optimization.

**Figure 18:**
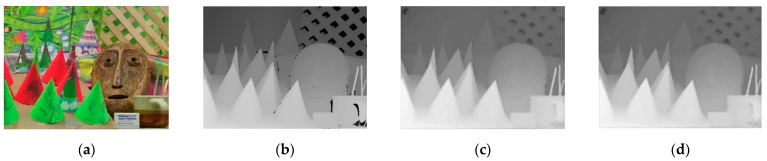
Scene of cones (**a**) RGB image; (**b**) Original depth image; (**c**) k=2; (**d**) k=4.

**Figure 19:**
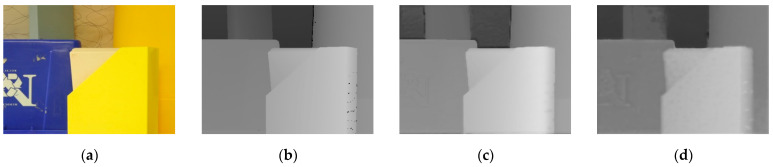
Scene of plastic (**a**) RGB image; (**b**) Original depth image; (**c**) k=2; (**d**) k=4.

Figure 18 and Figure 19 show the results of the depth map super-resolution tests for the Cones scene and the Plastic scene. These images are, in order: (a) the scene; (b) the original depth map; (c) the results at k = 2; and (d) the results at k = 4.

#### 5.2.3. Speed

The proposed algorithm does not need pre-processing, and all the processing is done by convolutional neural network, so it has a much better running speed compared with LRMC and DE-CNN. 1 min (0.017 fps) is needed for LRMC to process a 320×240 image, and 0.083 s (12.048 fps) is needed for DE-CNN including the pre-processing part. As shown in Table 9, it takes about 0.0372 s to process a Y-D image with a resolution of 320×240 on a single GTX1080, which translates into a frame rate of 25 fps, a 1470-fold and 2-fold improvement compared with LRMC and DE-CNN; while a Y-D image with a resolution of 640×480 can reach 13 fps. The relationship between speed and resolution is not linear, mainly because the CuDNN library uses some efficient algorithms such as Winograd to speed up the computation when implementing large-scale convolution operations.

TensorRT further optimizes the computation of convolution on top of CuDNN, and can fuse some eligible layers together to reduce the number of scheduling, maximizing the computational power of GPU and improving the efficiency of video memory usage. Since we do not quantize the weights (we still use 32-bit floating point numbers), the optimization strategy used by TensorRT for the network structure of this paper is mainly vertical layer fusion, which includes the following three optimizations: (1)The four operations of convolution, bias, batch normalization and ReLU in the first class of convolutional layers are fused into one CBR kernel; (2)The two operations of convolution and bias in the second class of convolutional layers are fused into one CBR kernel; (3) Canceling Concat layer by pre-allocating cache.

When the TensorRT framework is not adopted, the data scheduling and computational resource allocation among the Conv, Bias, BatchNorm, Scale and ReLU layers in the first class of convolutional layers will take up a lot of time. This non-computational time overhead is greatly reduced with the TensorRT framework. Note that since TensorRT 4 does not support slice layers, we have to replace the slice layers with two input layers to input the depth map and the intensity map.

With the TensorRT framework deployment, a single GTX1080 processing speed can achieve 320×240@47fps and 640×480@38fps, which are 1.88 times and 2.92 times faster than the speed without the TensorRT framework. The whole processing process can be divided into two parts: the IO phase between host memory and GPU memory, and the GPU computation phase, and our time consumption statistics for these two parts are shown in Table 10 and Table 11.

Finally, we tested the acquisition speed using C++ Streamer, a speed measurement software provided by Cypress. The result is stable at 50,400 KBps = 1,720,320 Bytes × 30 fps, which shows that the frame rate control of this design is very accurate. Assuming full speed operation at 60 MHz bus clock without frame rate control, the upload speed can be stabilized at 231,500 KBps = 226 MBps. The maximum frame rate of AR0135CS is 54 fps at 1280×960, which means the acquisition system can fully support the camera running at full speed.

### 5.3. Result of Practical Test

**Figure 20:**
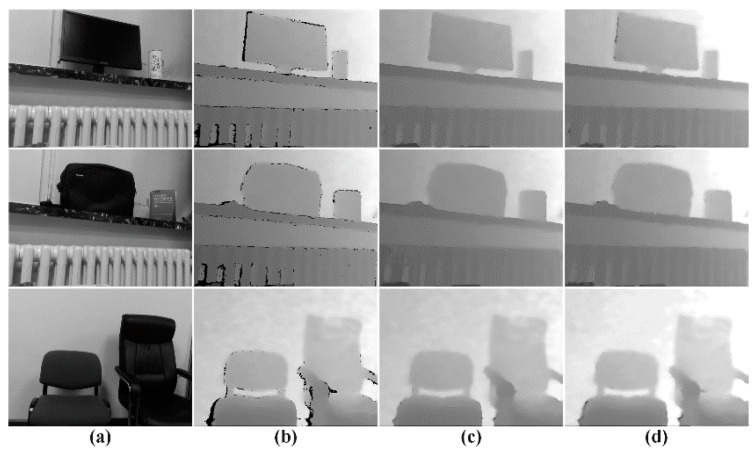
Results of practical test (**a**) Intensity image; (**b**) Depth image; (**c**) k=2; (**d**) k=4.

The practical test is shown in Figure 20 and we also calculated the PSNR, RMSE and SSIM, which are listed in Table 12, and these data basically match with the previous simulation results.

## 6. Conclusions

In this paper, we presented the design and implementation of a real-time depth map enhancement system based on residual network. A depth map enhancement algorithm is proposed, extracting features from the acquired intensity map and depth map in two ways, and then fusion and residual calculation are performed. Besides, the algorithm proposed adopts a full convolutional network and eliminates the pre-processing process. On a single GTX1080 graphics card, the processing speed can reach 320×240@25fps or 640×480@13fps without the TensorRT framework, which is 1470 times and 2 times faster than LRMC and DE-CNN respectively; and the speed is further increased to 320×240@47fps or 640×480@38fps with the TensorRT framework. Moreover, a FPGA-based dual-camera synchronous real-time acquisition system is implemented, which can precisely control ToF camera and visible camera to acquire images synchronously and can transfer the data to the back-end acceleration platform in real time via USB 3.0 interface. The experimental results show that the data throughput of the acquisition system is stable at 226 Mbps, and support dual-camera to work at full speed.

## Figures and Tables

**Table 1:** Configurations of asynchronous FIFOs.

	8 Byte FIFO	16 Byte FIFO
Read bit wide	32	32
Read depth	512	512
Write bit wide	8	16
Write depth	2048	1024
Read mode	First-word Fall-through	First-word Fall-through
storage medium	18 Kb BRAM × 1	18 Kb BRAM × 1

**Table 2:** Resources utilization.

Resources	Utilization	Total	Percentage (%)
LUT	4214	1,182,240	0.36
LUTRAM	215	591,840	0.04
FF	4006	2,364,480	0.17
BRAM	18	2160	0.83
URAM	210	960	21.88
IO	101	832	12.14
BUFG	8	1800	0.44
MMCM	3	30	10.00

**Table 3:** Parameters of the image sensors in this paper.

	AR0135CS	OPN8008
Working mode	Trigger mode	Trigger mode
IO level standard	1.8 V	3.3 V
Pixel Data Dimensions	1280×960	328×248×3
Pixel data format	12 bit Monochrome	RAW12
Sensor Configuration Bus	I^2^C Bus	I^2^C Bus
Pixel Data Transfer Bus	Parallel Interface	MIPI CSI-2

**Table 4:** Original PSNR performance (dB).

	Bowling 1	Bowling 2	Cloth 1	Cloth 2	Cloth 3	Cloth 4
5% holes	18.598333	18.013552	20.250878	17.642017	20.801490	18.257972
10% holes	15.582313	15.031216	17.259421	14.635192	17.795489	15.259041
15% holes	13.813898	13.294900	15.499276	12.873909	16.028865	13.499192
20% holes	12.555023	12.036268	14.237644	11.618691	14.772910	12.242208

**Table 5:** PSNR performance (dB) after inpainting.

	Bowling 1	Bowling 2	Cloth 1	Cloth 2	Cloth 3	Cloth 4
5% holes	42.462463	42.173859	47.267376	44.200977	43.456047	44.102913
10% holes	42.732605	42.454369	48.292862	44.581429	44.111671	43.971207
15% holes	42.717278	42.260109	48.252022	44.728699	44.343636	44.061874
20% holes	42.735268	41.923935	47.750134	44.527683	44.169636	43.622093

**Table 6:** Depth inpainting comparison (dB).

	Bowling 1	Bowling 2	Cloth 1	Cloth 2	Cloth 3	Cloth 4
LRMC	38.22	39.37	46.24	42.35	42.37	37.43
DE-CNN	37.52	38.71	45.01	41.45	42.75	39.16
DDTF [12]	38.00	38.18	46.86	42.33	42.16	37.57
Method proposed	**42.67**	**42.30**	**48.35**	**44.71**	**44.28**	**44.06**

**Table 7:** Depth super-resolution comparison, k=2.

	Aloe	Baby	Cones	Plastic	Teddy	Venus
Paper [45]	4.93	3.26	4.08	3.16	3.18	1.92
Paper [44]	2.89	1.81	2.13	1.81	1.74	0.98
Method proposed	**0.13**	**0.065**	**0.17**	**0.071**	**0.16**	**0.047**

**Table 8:** Depth super-resolution comparison, k=4.

	Aloe	Baby	Cones	Plastic	Teddy	Venus
Paper [45]	7.29	4.49	5.88	3.31	4.53	1.89
Paper [44]	5.12	2.97	3.73	2.63	2.86	1.67
Method proposed	**0.17**	**0.12**	**0.23**	**0.14**	**0.21**	**0.11**

**Table 9:** Elapsed time (s) without TensorRT framework.

	Bowling 1	Bowling 2	Cloth 1	Cloth 2	Cloth 3	Cloth 4
Elapsed time (s)	0.0372044	0.0375206	0.0374086	0.0372178	0.03684	0.0374244

**Table 10:** Time consumption of each phase using TensorRT under 320×240 resolution.

	IO Phase	Computing Phase	Total
Elapsed time (s)	0.000442	0.020668	0.021110
Percentage (%)	2.094	97.906	100

**Table 11:** Time consumption of each phase using TensorRT under 640×480 resolution.

	IO Phase	Computing Phase	Total
Elapsed time (s)	0.002566	0.023228	0.025794
Percentage (%)	9.948	90.052	100

**Table 12:** Resources utilization.

	PSNR (dB)	RMSE	SSIM
Scenario 1	42.449310	0.182983	0.949597
Scenario 2	41.113400	0.157987	0.954358
Scenario 3	41.554905	0.201898	0.933705

## Data Availability

Publicly available datasets were analyzed in this study. This data can be found here: Caffe: [https://caffe.berkeleyvision.org/tutorial/layers/hdf5data.html, accessed on 16 December 2020], Middlebury: [http://graphics.cs.pdx.edu/project/depth-enhance/, accessed on 16 December 2020], MPI Sintel [http://sintel.is.tue.mpg.de/, accessed on 16 December 2020].
